# Supplementary material for: Causal Relationship between Aging and Anorexia Nervosa: A White-Matter-Microstructure-Mediated Mendelian Randomization Analysis
Source: Biomedicines. 2024 Aug 16;12(8):1874. doi: 10.3390/biomedicines12081874 (PMC11351342; doi:10.3390/biomedicines12081874)
Supplement: Supplementary file 1 [file biomedicines-12-01874-s001.zip › Supplementary Figure/Figure S9/Leaveoneout plot of MR analysis for ubm-b-1474 to AN.pdf]

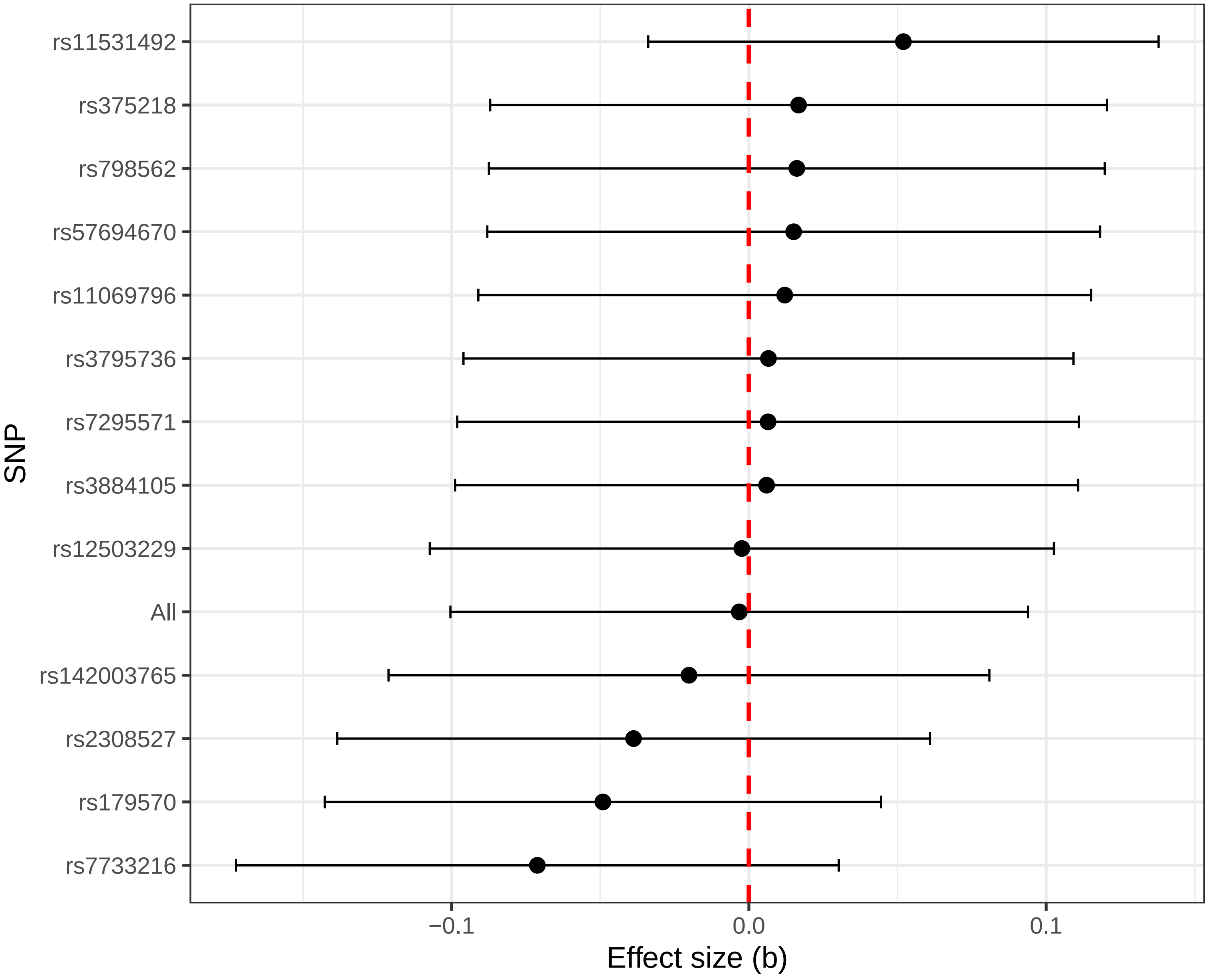
rs11531492
rs375218
rs798562
rs57694670
rs11069796
rs3795736
rs7295571
rs3884105
rs12503229
All
rs142003765
rs2308527
rs179570
rs7733216
SNP
−0.1
0.0
0.1
Effect size (b)
